# Plasma concentration and expression of adipokines in epicardial and subcutaneous adipose tissue are associated with impaired left ventricular filling pattern

**DOI:** 10.1186/s12967-019-2060-7

**Published:** 2019-09-18

**Authors:** Kacper Toczylowski, Tomasz Hirnle, Dorota Harasiuk, Piotr Zabielski, Anna Lewczuk, Iwona Dmitruk, Monika Ksiazek, Artur Sulik, Jan Gorski, Adrian Chabowski, Marcin Baranowski

**Affiliations:** 1 Department of Pediatric Infectious Diseases, Medical University of Bialystok, Waszyngtona 17, 15-274 Bialystok, Poland; 2 Department of Cardiac Surgery, Medical University of Bialystok, Sklodowskiej 24A, 15-276 Bialystok, Poland; 3 Department of Physiology, Medical University of Bialystok, Mickiewicza 2C, 15-222 Bialystok, Poland

**Keywords:** Epicardial adipose tissue, Obesity, Type 2 diabetes mellitus, Heart failure, Adipokines, Leptin, Adiponectin, Apelin, Resistin, Visfatin

## Abstract

**Background:**

Adipokines in serum derive mainly from subcutaneous and visceral adipose tissues. Epicardial adipose tissue (EAT), being a relatively small but unique fat depot, probably does not make an important contribution to systemic concentrations of adipokines. However, proximity of EAT to cardiac muscle and coronary arteries allows cells and proteins to penetrate between tissues. It is hypothesized that overexpression of proinflammatory cytokines in EAT plays an important role in pathophysiology of the heart. The aim of the study was to analyze the relationship between echocardiographic heart parameters and adipokines in plasma, epicardial, and subcutaneous fat in patients with obesity and type 2 diabetes mellitus (T2DM). Additionally, we evaluate proinflammatory properties of EAT by comparing that depot with subcutaneous adipose tissue.

**Methods:**

The study included 55 male individuals diagnosed with coronary artery disease (CAD) who underwent planned coronary artery bypass graft. Plasma concentrations of leptin, adiponectin, resistin, visfatin, apelin, IL-6, and TNF-α, as well as their mRNA and protein expressions in EAT and subcutaneous adipose tissue (SAT) were determined.

**Results:**

Obesity and diabetes were associated with increased leptin and decreased adiponectin plasma levels, higher protein expression of leptin and IL-6 in SAT, and higher visfatin protein expression in EAT. Impaired left ventricular (LV) diastolic function was associated with increased plasma concentrations of leptin, resistin, IL-6, and adiponectin, as well as with increased expressions of resistin, apelin, and adiponectin in SAT, and leptin in EAT.

**Conclusions:**

Obesity and T2DM in individuals with CAD have a limited effect on adipokines. Expression of adipokines in EAT and SAT is linked to certain heart parameters, however diastolic dysfunction of the LV is strongly associated with circulating adipokines.

## Background

The discovery of leptin in 1994 was a huge step towards understanding that adipose tissue is something more than just an energy storage [1]. Soon after the discovery numerous proteins secreted from adipose tissue, named adipokines, were described. It has been established that human homeostasis is heavily influenced by adipose tissue which serves as a paracrine and endocrine organ, and produces a wide array of adipokines. Adipokines are known to induce insulin resistance, systemic inflammation, hypercoagulability, and endothelial dysfunction, all of which promote atherosclerosis in individuals with obesity and diabetes [2]. Adipose tissue distribution appears to be an important contributor to adipokine levels and therefore, to obesity-related diseases. Recently more research was focused on epicardial adipose tissue (EAT) because of its different metabolism and unique anatomy.

EAT surrounds atria, right ventricular free wall, apex of the left ventricle, and major branches of coronary arteries [3]. Its mass is relatively small and it probably does not make a significant contribution to systemic adipokine concentrations, as opposed to much bigger visceral and subcutaneous fat depots. However, EAT shares blood supply with cardiac muscle, and there are no anatomic structures separating both tissues, allowing cells and proteins to cross between the tissues. EAT may take an important part in pathophysiology of heart disease through production of both cardioprotective and pro-inflammatory adipokines that have paracrine and vasocrine effects on the myocardium [4, 5]. However, the exact role of EAT in heart disease in patients with obesity and type 2 diabetes mellitus (T2DM) remains unclear mainly because data on adipokine expression in EAT is scarce.

Of the large numbers of known adipokines and cytokines, several are believed to play a key role in obesity-related diseases. Leptin levels correlate with body fat mass and provide feedback to inform hypothalamus about energy reserves. Although leptin levels in obesity are usually high, a lack of satiation is observed indicating a state of leptin resistance. High leptin levels still have wide-ranging effects on lipid and carbohydrate metabolism, angiogenesis, and cardiovascular, immune and reproductive systems [6]. Resistin, interleukin 6 (IL-6), and tumor necrosis factor α (TNF-α) trigger inflammatory response. By targeting macrophages, peripheral blood mononuclear cells and vascular cells, these cytokines contribute to progression of atherosclerosis and cardiovascular disease [7–9]. On the contrary, adiponectin and apelin seem to protect from the development of obesity-related diseases by exerting an anti-oxidant activity, reducing inflammation, preventing cell apoptosis and limiting fibrosis [10, 11]. Visfatin is linked with lipid and carbohydrate metabolism, its increased expression was found in foam cells from atherosclerotic lesions. The exact role of visfatin in obesity remains elusive [12].

Given the above, the aim of the present study was to analyze the relationship between expression of adipokines in epicardial and subcutaneous adipose tissue (SAT), and morphological and functional parameters of the heart in patients with obesity and T2DM. Additionally, to assess proinflammatory properties of EAT, we compare profiles of adipokines in EAT and SAT.

## Methods

### Study design and participants

The study included 55 male individuals diagnosed with coronary artery disease (CAD) who underwent planned coronary artery bypass graft at the Department of Cardiac Surgery, Medical University of Bialystok, Poland. Exclusion criteria were cardiac arrhythmias, heart valve disease, myocardial infarction in the last 30 days, chronic morbidity (other than type 2 diabetes, hypertension or hyperlipidemia), additional cardiac surgery that was not planned beforehand, age over 80 years or under 30, body mass index (BMI) over 40 kg/m^2^. Patients were divided into three groups: I. The non-obese controls, without obesity and diabetes mellitus, with BMI < 26 kg/m^2^, normal fasting blood glucose (< 100 mg/dL) and normal glycated hemoglobin (< 6%) (n = 14); II. patients with overweight or obesity (BMI > 26 kg/m^2^) without diabetes mellitus, with normal fasting blood glucose and normal glycated hemoglobin (n = 27); III. patients with overweight or obesity (BMI > 26 kg/m^2^) and with T2DM (n = 14). The study was approved by the Ethics Committee of Medical University of Bialystok (decision number R-I-002/358/2010) and adhered to the principles of the Declaration of Helsinki. All the individuals agreed to participate in the study and gave informed consent.

### Clinical assessments

In all patients an assessment of clinical and anthropometrical parameters was recorded. On the day of the surgery, a fasting blood sample was collected to measure serum glucose, insulin, triacylglycerol, total cholesterol, HDL-cholesterol and LDL-cholesterol concentrations. An evaluation of morphological and functional heart parameters by transthoracic echocardiography was done before the surgery, including two-dimensional, M-mode and Doppler imaging. EAT thickness was measured on the free wall of the right ventricle (RV) from both parasternal long- and short-axis views [13]. Right after chest opening, samples of subcutaneous adipose tissue from the sternal region and epicardial adipose tissue adjacent to the right coronary artery were collected, snap frozen in liquid nitrogen, and then stored in − 80 °C upon further analysis.

### Laboratory analysis

Plasma adipokine concentrations were measured using enzyme-linked immunosorbent assays (ELISA). Leptin concentrations were assessed with Human Leptin ELISA kit (Merck Millipore, MA, US), adiponectin with Human Adiponectin Elisa kit (Merck Millipore, MA, US), resistin with Human Resistin Elisa kit (Merck Millipore, MA, US). IL-6 was measured with IL-6 High Sensitivity ELISA kit (eBioscience, CA, US), TNF-α with Human TNF-α High Sensitivity ELISA kit (eBioscience, CA, US). Apelin levels were measured using Apelin-36 (Human)—EIA kit (Phoenix Pharmaceuticals, CA, US), and visfatin using Enzyme-linked Immunosorbent Assay Kit For Visfatin kit (Uscn Life Science Inc, TX, US).

Adipose tissue samples for analysis of adipokine expression were pulverized and reduced with crystalline Tris(2-carboxyethyl)phosphine hydrochloride (TCEP). Then, RNA and protein were isolated using NucleoSpin RNA/Protein kit (Macherey–Nagel, Germany).

Adipokine mRNA expression in adipose tissue samples was measured by real-time polymerase chain reaction. First, using High Capacity cDNA Reverse Transcription Kit (Applied Biosystems, Thermo Fisher Scientific, MA, US) a reverse transcriptase PCR was undertaken. Then, in thermocycler Chromo4 (Bio-Rad Laboratories, CA, US) using SYBR Green JumpStart Taq ReadyMix (Sigma-Aldrich, MO, US) a real-time PCR-amplification was performed. Forward and reverse primers are listed in Table 1. Cyclophilin A was used as a housekeeping gene [14].

**Table 1:** Forward and reverse primers used in real-time PCR

Gene	Forward primer	Reverse primer
Resistin	5′-TCT AGC AAG ACC CTG TGC-3′	5′-TGC TTA TTG CCC TAA ATA TTA G-3′
Adiponectin	5′-AGT CTG TGG TTC TGA TTC C-3′	5′-TTG AGT CGT GGT TTC CTG-3′
Leptin	5′-TTT CAC ACA CGC AGT CAG-3′	5′-CCA TCT TGG ATA AGG TCA GG-3′
IL-6	5′-CTG GAT TCA ATG AGG AGA CTT G-3′	5′-CTC ACT ACT CTC AAA TCT GTT CTG-3′
Visfatin	5′-CGG TTC TGG TGG AGG TTT GC-3′	5′-CCT GCT GGC GTC CTA TGT AAA G-3′
TNF-α	5′-GGG AGC CTT TGG TTC TGG-3′	5′-AGG AAG TCT GGA AAC ATC TGG-3′
Apelin	5′-GCT CTC ACC TCG CAC CTG-3′	5′-GAT GGA CTG GAC GGA TTC TTG-3′
CYCA	5′-ATC CTA GAG GTG GCG GAT TT-3′	5′-CAC TCA GGT CTG AGC CAC AA-3′

*IL-6* interleukin 6, *TNF-α* tumor necrosis factor α, *CYCA* cyclophilin A

To analyze protein expression of adipokines, Western blots were performed using lysates from tissues. First, an electrophoresis was performed in TGS buffer (BioRad), in which Precision Plus Protein Western C Standards (BioRad) were used. Then the proteins were transferred to polyvinylidene difluoride transfer membrane with semi-dry technique and incubated with primary antibodies: leptin (Abcam, Cambridge, UK, cat. no ab9826), adiponectin (Abcam, cat. no. ab75989), TNF-α (Abcam, cat. no. ab66579), IL-6 (Abcam, cat. no. ab93356), resistin (Abcam, cat. no. ab124681), apelin (Abcam, cat. no. ab125213). As a secondary antibody a goat anti-rabbit IgG-HRP was used (Santa Cruz Biotechnology, cat. no. sc-2004). Bands were visualized by chemiluminescence (Molecular Imager ChemiDoc XRS+, BioRad). Expression of adipokines was normalized to GAPDH (GAPDH Antibody, Santa-Cruz Biotechnology, cat. no. sc-25778) protein levels for each sample.

### Statistical analysis

Statistical analysis was done using Statsoft Statistica version 12. Data are presented as mean ± standard deviation (SD). Differences between the groups were analyzed with one-way analysis of variance (ANOVA) followed by Newman-Keuls post hoc test. Non-parametric tests were used for other analyses. For analysis of the relationship between expression of adipokines and echocardiographic heart parameters, a stepwise regression analysis was utilized. The *p*-value below 0.05 was considered significant.

## Results

### Study group characteristics

Baseline characteristics of the study groups are presented in Table 2. Importantly, there were no significant differences in patients’ age, disease status, or drug use. Non-T2DM patients with obesity presented increased Homeostasis Model Assessment-Insulin Resistance (HOMA2-IR), but their glycated hemoglobin (HbA1_c_) and serum glucose levels were normal. The presence of diabetes was associated with increased waist-to-hip ratio, hyperglycemia, hypertriglyceridemia, and high HbA1_c_.

**Table 2:** Clinical characteristics, disease status and drug usage of the patients

	Controls (n = 14)	Obesity (n = 27)	Obesity + T2DM (n = 14)
Age (years)	59.6 ± 12.1	61.6 ± 9.6	62.9 ± 11.2
Height (cm)	172 ± 7	171 ± 5	172 ± 5
Weight (kg)	71.0 ± 7.8	86.6 ± 10.9^†^	90.3 ± 8.2^†^
Hip circumference (cm)	96.5 ± 6.2	109.0 ± 8.3^†^	106.9 ± 7.5^†^
Waist circumference (cm)	91.6 ± 6.3	106.0 ± 8.5^†^	109.5 ± 8.0^†^
Waist-to-hip ratio	0.95 ± 0.06	0.97 ± 0.04	1.03 ± 0.06^†,‡^
BMI (kg/m^2^)	24.0 ± 1.9	29.4 ± 3.0^†^	30.6 ± 2.7^†^
SBP (mmHg)	124 ± 13	128 ± 13	132 ± 13
DBP (mmHg)	72 ± 6	75 ± 7	74 ± 6
Disease status			
Hypertension, n (%)	10 (71%)	22 (81%)	12 (86%)
Dyslipidemia, n (%)	7 (50%)	17 (63%)	7 (50%)
NYHA [I/II/III/IV]	1/12/1/0	3/24/0/0	2/10/2/0
CCS [I/II/III/IV]	1/11/2/0	2/23/2/0	2/9/3/0
HFrEF, n (%)	11 (79%)	12 (44%)	6 (43%)
HFpEF, n (%)	2 (14%)	14 (52%)	7 (50%)
Biochemistry			
Glucose (mg/dL)	80.5 ± 6.1	86.7 ± 7.1	104.1 ± 24.4^†,‡^
Insulin (mU/L)	5.34 ± 2.35	8.94 ± 4.75^†^	8.44 ± 3.34^†^
HOMA2-IR	0.77 ± 0.33	1.30 ± 0.70^†^	1.31 ± 0.57^†^
HbA1_c_ (%)	5.77 ± 0.40	5.66 ± 0.48	7.61 ± 1.39^†,‡^
Triglycerides (mg/dL)	121 ± 72	146 ± 66	226 ± 197^†^
Total cholesterol (mg/dL)	175 ± 43	169 ± 51	155 ± 47
HDL-cholesterol (mg/dL)	37.6 ± 9.3	35.2 ± 6.3	34.1 ± 11.5
LDL-cholesterol (mg/dL)	114 ± 39	106 ± 45	83 ± 38
Drugs			
Beta-blockers, n (%)	12 (86%)	24 (89%)	12 (86%)
Angiotensin-converting-enzyme inhibitor, n (%)	13 (93%)	23 (85%)	13 (93%)
Statins, n (%)	10 (71%)	25 (93%)	12 (86%)
Metformin, n (%)	0	0	7 (50%)
Sulfonyloureas, n (%)	0	0	7 (50%)
Insulin, n (%)	0	0	5 (36%)

*BMI* body mass index, *SBP* systolic blood pressure, *DBP* diastolic blood pressure, *HFrEF* heart failure with a reduced ejection fraction, *HFpEF* heart failure with a preserved ejection fraction, *HOMA2-IR* Homeostasis model assessment of insulin resistance

^†^p < 0.05 as compared to non-obese controls

^‡^p < 0.05 as compared to patients with obesity

### Echocardiographic examination of heart structure and function

Results of echocardiographic examination are summarized in Table 3. Importantly, EAT thickness in non-obese controls was lower than in patients with obesity, but the differences were insignificant. Both systolic and diastolic heart function, as well as all heart structure parameters but left atrium diameter (LA), including left ventricular mass (LVM), left ventricular mass index (LVMi), interventricular septum diastolic diameter (IVSD), relative wall thickness (RWT), and posterior wall thickness in diastole (PWTd) were similar in analyzed groups.

**Table 3:** Echocardiographic parameters of the patients

	Controls	Obesity	Obesity + T2DM
EF (%)	47.7 ± 9.0	51.7 ± 9.7	53.4 ± 10.1
FS (%)	26.5 ± 7.7	33.2 ± 8.8	28.9 ± 7.1
TAPSE (mm)	25.9 ± 5.7	26.1 ± 4.1	24.0 ± 4.6
E/A	0.9 ± 0.3	0.8 ± 0.3	0.9 ± 0.4
DT (ms)	255.8 ± 86.9	227.6 ± 48.1	234.2 ± 64.7
IVRT (ms)	111.8 ± 21.2	107.9 ± 17.8	99.0 ± 19.6
E’ (cm/s)	8.0 ± 2.5	7.7 ± 1.7	7.0 ± 2.1
A’ (cm/s)	8.3 ± 1.9	8.7 ± 2.1	8.9 ± 1.5
DT’ (ms)	167.8 ± 63.3	165.5 ± 48.9	162.4 ± 57.3
E’/A’	1.1 ± 0.5	0.9 ± 0.3	0.8 ± 0.3
RVAC (mL)	43.9 ± 15.7	40.6 ± 9.5	39.8 ± 10.2
EATt (mm)	9.9 ± 2.2	12.1 ± 3.6	11.7 ± 2.9
LA (cm)	4.0 ± 0.4	4.1 ± 0.4	4.4 ± 0.5^†^
LVdD (cm)	5.4 ± 0.7	5.5 ± 0.5	5.4 ± 0.5
LVsD (cm)	3.9 ± 0.8	3.6 ± 0.6	3.9 ± 0.5
RVdD (cm)	2.4 ± 0.4	2.7 ± 0.4^†^	2.6 ± 0.4
IVSD (cm)	1.1 ± 0.2	1.1 ± 0.2	1.2 ± 0.2
PWTd (cm)	1.1 ± 0.1	1.1 ± 0.1	1.2 ± 0.2
RWT	0.4 ± 0.1	0.4 ± 0.1	0.4 ± 0.1
LVM (g)	226.8 ± 61.5	251.8 ± 58.6	260.0 ± 62.7
LVMi (g/m^2.7^)	52.9 ± 14.5	58.4 ± 11.8	59.9 ± 12.6

*EF* ejection fraction, *FS* fractional shortening, *TAPSE* tricuspid annular plane systolic excursion, *E/A* the ratio of peak velocity blood flow in early diastole to peak velocity flow in late diastole, *DT* deceleration time of mitral inflow velocity, *IVRT* isovolumic relaxation time, *E*′ peak mitral annulus velocity in early diastole, *A*′ peak mitral annulus velocity in late diastole, *DT*′ deceleration time of the mitral annulus measured in tissue Doppler, *RVAC* right ventriculo-arterial coupling, *EATt* epicardial adipose tissue thickness, *LA* left atrium diameter, *LVdD* left ventricular end-diastolic diameter, *LVsD* left ventricular end-systolic diameter, *RVdD* right ventricular end-diastolic diameter, *IVSD* interventricular septum thickness, *PWTd* posterior wall thickness at end-diastole, *RWT* relative wall thickness, *LVM* left ventricular mass, *LVMi* left ventricular mass index

^†^Indicates p < 0.05 as compared to non-obese controls

Notably, there were no significant differences in the frequency of heart failure (HF) revealed by clinical and echocardiographic examination. The majority of patients in each study group was classified in class II of the New York Heart Association (NYHA) and class II in The Canadian Cardiovascular Society (CCS) Functional Classification. None of the patients was presented with symptoms of HF at rest. In 41 (69.5%) patients a left ventricular hypertrophy (LVH) was diagnosed, as defined by LVMi ≥ 51 g/m^2.7^ [15]. LVH was detected in 7 (50%) non-obese controls, 22 (81%) patients with obesity, and 9 (64%) patients with obesity and diabetes. Importantly, impaired left ventricle (LV) relaxation, as defined by decrease in the ratio of peak velocity blood flow in early diastole to peak velocity flow in late diastole caused by atrial contraction (E/A ratio) under 0.8, was observed in 7 non-obese controls (50%), 13 patients with obesity (48%), and 9 patients with obesity and diabetes (64%) patients. None of the patients presented pseudonormal LV filling pattern. In patients with obesity and diabetes there was only one individual with restrictive filling pattern described by E/A = 2.25. Six non-obese controls (43%), 13 patients with obesity (48%), and 3 patients with obesity and diabetes (21%) had E/A ratio between 0.8 and 1.5, but presented signs of impaired LV filling, including prolonged deceleration time (DT), prolonged isovolumic relaxation time (IVRT) and/or increased LA. One patient in each study group did not meet echocardiographic criteria for heart failure, but all three presented clinical signs of heart failure.

### Plasma adipokine concentration

**Fig. 1:**
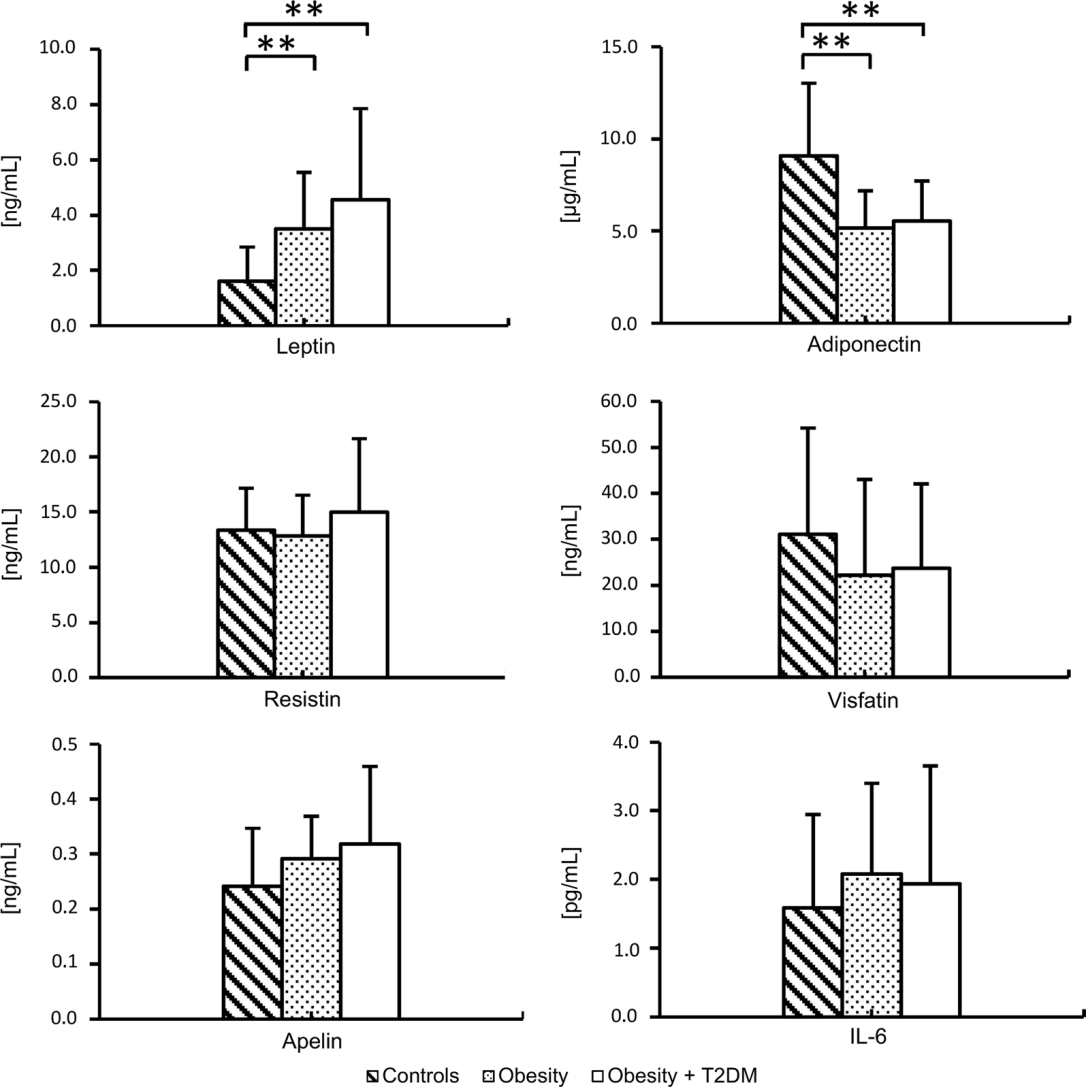
Concentration of adipokines in plasma measured by ELISA (n = 55); differences between the groups were analyzed with one-way analysis of variance (ANOVA) followed by Newman-Keuls post hoc test; **indicates p < 0.01

Decreased plasma levels of adiponectin, but increased levels of leptin were found in patients with obesity (Fig. 1). Concentrations of resistin, visfatin, IL-6 and apelin were similar across the groups. TNF-α was detected in only 9 plasma samples (17%), therefore it was not included in the comparison.

### Expression of adipokines in SAT and EAT depots

**Fig. 2:**
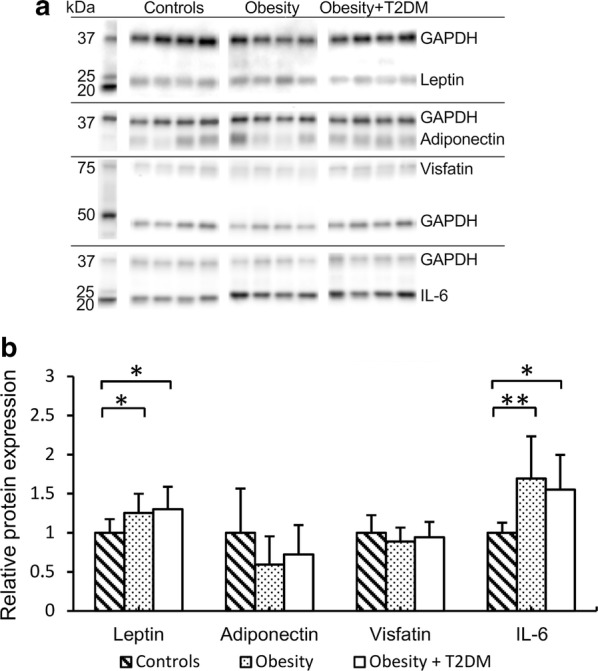
Relative protein expression of adipokines in subcutaneous fat tissue depots measured by Western blot (n = 33). Representative blots for each protein are shown in **a**. **b** shows relative protein levels. Bands were visualized by chemiluminescence; GAPDH was used as a loading control. The Mann–Whitney U test was used to determine differences between fat depots; *indicates p < 0.05; **indicates p < 0.01; IL-6, interleukin 6; GAPDH, Glyceraldehyde 3-phosphate dehydrogenase

**Fig. 3:**
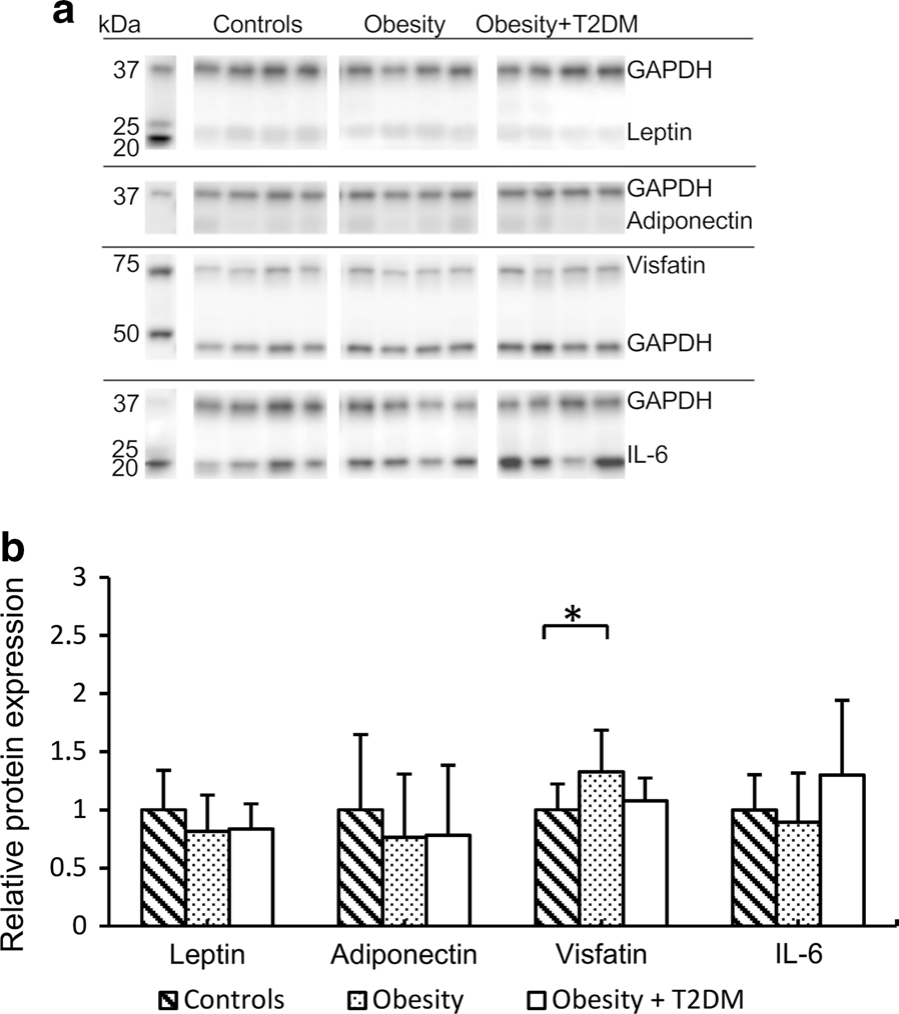
Relative protein expression of adipokines in epicardial fat tissue depots measured by Western blot (n = 33). Representative blots for each protein are shown in **a**. **b** shows relative protein levels. Bands were visualized by chemiluminescence; GAPDH was used as a loading control. The Mann–Whitney U test was used to determine differences between fat depots; *indicates p < 0.05; **indicates p < 0.01; IL-6, interleukin 6; GAPDH, glyceraldehyde 3-phosphate dehydrogenase

Messenger RNA expression of all seven adipokines did not differ between the groups in SAT and EAT fat depots (data not shown). Protein expression of TNF-α, apelin and resistin was below the detection threshold in both fat depots. In patients with obesity and diabetes there was approximately 1.3-fold higher protein expression of leptin, and ~ 1.7-fold higher protein expression of IL-6 in SAT, as compared to non-obese controls. Visfatin protein expression in EAT was 1.3-fold higher in patients with obesity than in non-obese controls (Figs. 2 and 3). Protein expression of other adipokines in SAT and EAT depots did not differ between the groups. The presence of diabetes did not influence adipokine protein expression in either adipose tissue depot in patients with obesity.

### Comparison of adipokine mRNA and protein expression in SAT and EAT depots

**Fig. 4:**
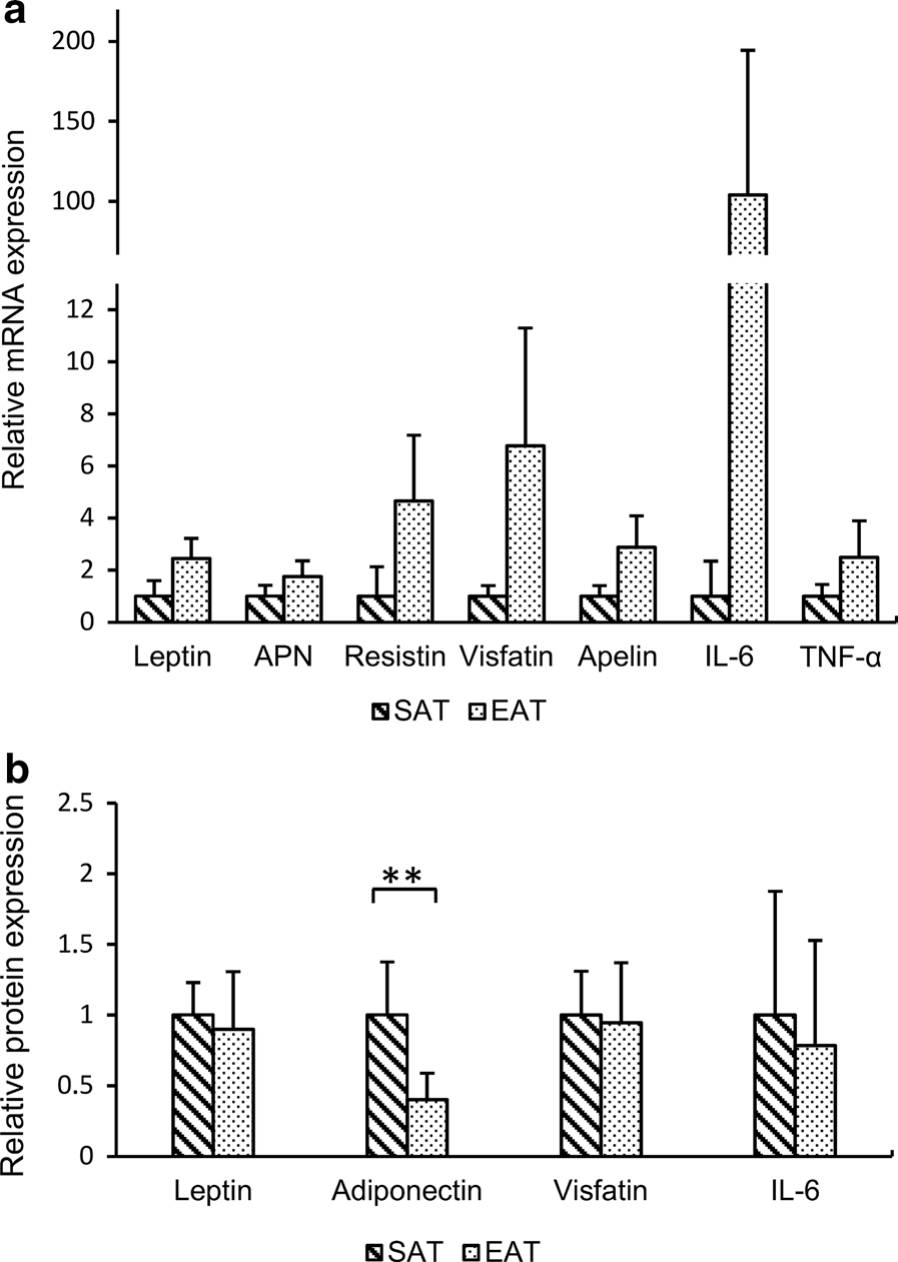
Relative mRNA (**a**) and protein (**b**) expression of adipokines in the SAT and EAT samples. PCR was performed in 55 patients, for Western blots 4 patients in each study group were selected randomly (12 in total). Cyclophillin A was used as a housekeeping gene in PCR, GAPDH was a loading control in Western blots. Quantifications in PCR were made with a Pfaffl method. The Mann–Whitney U test was used to determine differences between fat depots. Each comparison of mRNA expression was statistically significant with *p* < 0.001; **indicates *p* < 0.001 in protein expression. *EAT* epicardial adipose tissue, *SAT* subcutaneous adipose tissue, *APN* adiponectin, *IL-6* interleukin 6, *TNF-α* tumor necrosis factor α, *GAPDH* glyceraldehyde 3-phosphate dehydrogenase

Messenger RNA expression of adipokines in fat depots was compared in the cohort of all patients. We found significantly higher gene expression of all adipokines in EAT than in SAT depot. We report 1.8-fold higher expression of adiponectin, 2.4-fold higher mRNA expression of leptin, 2.5-fold higher expression of TNF-α, 2.9-fold higher expression of apelin, 4.6-fold higher expression of resistin, 6.8-fold higher expression of visfatin, and 104.2-fold higher expression of IL-6 in the EAT, compared to the SAT depot (*p *< 0.001 for all comparisons) (Fig. 4a). The comparison of protein expression was done in a separate Western blot analysis of 12 patients, 4 randomly selected from each study group. Similarly to the first analysis, protein expression of TNF-α, apelin and resistin was below the detection threshold in both fat tissue depots. Therefore only leptin, adiponectin, visfatin and IL-6 protein expressions were analyzed. We found that the content of adiponectin protein in EAT was only 40% of that in SAT (*p *< 0.001). The protein expression of other adipokines did not differ (Fig. 4b). For representative blots see Additional file 1.

### Association between tissue expression and plasma concentration of adipokines and heart structure and function

We hypothesized that adipokines are associated with heart structure and function. A simple analysis of correlation would not give reliable results, because age and BMI have an impact on both heart parameters and adipokines. Therefore we created a regression model. In the model we included three dependent variables: plasma concentration or tissue expression of an adipokine, patient’s age, and patient’s BMI. Then we performed a stepwise regression analysis in which we identified adipokines that were related to heart structure and function, independently of age and BMI. Results are presented in Table 4. Impaired LV diastolic function was associated with increased plasma concentration of leptin, resistin, IL-6, and adiponectin, as well as with elevated level of resistin mRNA, apelin mRNA, and adiponectin mRNA and protein in SAT, and leptin mRNA and protein in EAT. A moderate correlation was found between ejection fraction (EF) and apelin mRNA, IL-6 protein, and leptin protein expressions in SAT. Importantly, none of the plasma adipokines was related to LV systolic function. However, plasma IL-6 was related to TAPSE, a parameter describing systolic function of the RV. The relationship of adipokines and parameters describing heart size did not make a clear pattern. Visfatin protein expression in EAT was inversely related to LA, but correlated positively with PWTd. Protein expression of leptin in EAT correlated negatively with PWTd, the relation with IVSD was, however, positive. Messenger RNA expression of TNF-α in the SAT correlated positively with LVM and RVdD. EAT thickness correlated only with apelin mRNA expression in the SAT.

**Table 4:** Stepwise regression analysis of adipokines and echocardiographic heart parameters

	*β*	*p*
Systolic heart function		
EF		
Apelin SAT mRNA	− 0.29 ± 0.13	0.036
TNF-α EAT mRNA	− 0.36 ± 0.13	0.010
IL-6 SAT protein	0.38 ± 0.16	0.025
Leptin SAT protein	0.37 ± 0.16	0.033
TAPSE		
IL-6 plasma	− 0.34 ± 0.14	0.015
Diastolic heart function		
E/A		
Apelin plasma	0.40 ± 0.12	0.001
Resistin plasma	− 0.30 ± 0.12	0.017
Resistin SAT mRNA	− 0.36 ± 0.12	0.005
Leptin plasma	− 0.28 ± 0.13	0.033
DT		
Resistin plasma	0.38 ± 0.13	0.005
Adiponectin plasma	0.36 ± 0.13	0.007
Adiponectin SAT mRNA	0.31 ± 0.13	0.025
Adiponectin SAT protein	0.52 ± 0.15	0.002
Apelin SAT mRNA	0.34 ± 0.13	0.012
E′		
IL-6 plasma	− 0.28 ± 0.13	0.030
A′		
Leptin plasma	0.34 ± 0.16	0.041
Leptin EAT protein	0.49 ± 0.15	0.003
E′/A′		
IL-6 plasma	− 0.34 ± 0.13	0.014
IL-6 EAT protein	− 0.37 ± 0.17	0.033
DT′		
Leptin EAT mRNA	0.44 ± 0.13	0.002
Resistin SAT mRNA	0.35 ± 0.13	0.008
Heart structure		
EATt		
Apelin SAT mRNA	0.35 ± 0.14	0.014
PWTd		
Visfatin plasma	0.46 ± 0.13	0.001
Adiponectin plasma	− 0.37 ± 0.13	0.005
Leptin EAT protein	− 0.38 ± 0.16	0.021
RVdD		
Resistin plasma	0.39 ± 0.12	0.002
TNF-α SAT mRNA	0.27 ± 0.13	0.036
LA		
Visfatin EAT protein	− 0.38 ± 0.15	0.017
IVSD		
Leptin EAT mRNA	0.28 ± 0.13	0.041
LVM		
TNF-α SAT mRNA	0.35 ± 0.13	0.011

Stepwise regression analysis was performed on adipokines and parameters of heart function and heart structure; patient’s BMI and age were included in the model

*EF* ejection fraction, *TAPSE* tricuspid annular plane systolic excursion, *E/A* the ratio of peak velocity blood flow in early diastole to peak velocity flow in late diastole, *DT* deceleration time of mitral inflow velocity, *E*′ peak mitral annulus velocity in early diastole, *A*′ peak mitral annulus velocity in late diastole, *DT*′ deceleration time of the mitral annulus measured in tissue Doppler, *EATt* epicardial adipose tissue thickness, *PWTd* posterior wall thickness at end-diastole, *RVdD* right ventricle diameter in diastole, *LA* left atrium diameter, *IVSD* interventricular septum thickness, *LVM* left ventricular mass, *β* standardized regression coefficient

### Adipokine profile in patients with heart failure and preserved ejection fraction

Adipokine profiles between two types of heart failure were compared. Individuals with a preserved EF of the LV (HFpEF), compared to patients with a reduced EF (HFrEF), had 0.7-fold lower EAT expression of TNF-α mRNA (p = 0.027), but 1.7-fold higher EAT expression of IL-6 mRNA (p = 0.020) and 1.7-fold higher EAT expression of visfatin mRNA (p = 0.009). Plasma adiponectin concentration was lower in HFpEF (5.52 ± 3.13 vs. 6.92 ± 2.98 µg/mL; p = 0.024). Plasma concentrations and tissue expressions of other adipokines did not differ between the two types of HF. Interestingly, in individuals with HFpEF thickness of EAT was higher (12.5 ± 2.4 vs. 10.2 ± 3.5 mm; p = 0.021). Importantly, there were no differences regarding age, BMI, blood pressure, drug use or presence of comorbidities between patients with HFpEF and HFrEF.

## Discussion

Obesity and diabetes are known risk factors of heart disease [2]. Individuals with abdominal obesity more frequently present heart diseases than individuals with other types of obesity. It indicates that visceral fat is strongly related to heart pathologies [16]. Adipose tissue produces considerable amounts of adipokines, some of which exert effects on the heart muscle and arteries. Adipokine levels seem to be the key determinants of metabolic alterations in individuals with obesity [2].

### Adipokines in plasma and fat tissue samples

We report an increase in plasma leptin and a decrease in plasma adiponectin levels in patients with obesity or diabetes. Leptin protein content in SAT was also increased in individuals with obesity. We hypothesize that plasma leptin levels are, at least partly, a reflection of SAT leptin overexpression. High volumes of SAT in individuals with obesity add to that effect. Similarly to our results, Kouidhi et al. reported higher mRNA leptin levels in SAT in patients with obesity [17]. We also found increased IL-6 protein expression in SAT in individuals with obesity, what is consistent with other studies [18]. Moreover, an elevated visfatin protein, but not mRNA expression was noted in EAT in patients with obesity. Plasma concentrations and tissue expressions of other adipokines were not affected by obesity or T2DM. Possibly, underlying CAD heavily influenced adipokine concentrations and tissue expressions, and as a result we failed to demonstrate any changes caused by obesity and diabetes alone. It has been observed before, that CAD is associated with an increase in proinflammatory adipokines tissue content. CAD might even be described by a unique profile of cytokines in serum [19, 20].

### Comparison of SAT and EAT adipokine expression

A significantly higher mRNA expression of all adipokines, especially pro-inflammatory IL-6, was detected in the EAT, as compared to the SAT. Despite higher gene expression of adipokines, protein expressions of leptin, visfatin, and IL-6 were similar in the SAT and EAT. Moreover, despite higher adiponectin mRNA expression, its protein content in EAT was lower than in SAT. Previous studies have already demonstrated that EAT is an important source of proinflammatory cytokines [21–24]. In contrary to our results, Bambace et al. reported lower adiponectin mRNA levels in EAT than in SAT [25]. Decreased abundance of anti-inflammatory adiponectin in EAT in the presence of heavily overexpressed IL-6 that we observed could be associated with pathophysiology of CAD. However, these results should be interpreted with caution, because of the discrepancy between protein and mRNA expressions. That disparity could lie in the elaborate process of protein production. Protein abundance is regulated by post-transcriptional, translational and protein degradation processes [26]. Tissue mRNA levels were reported to explain only 40% of variability in tissue protein levels [27]. Therefore, gene and protein expressions may differ in tissues. The discrepancy observed in our study casts in doubt results of other studies performed on adipose tissue where only mRNA expressions were investigated. Nevertheless, we clearly demonstrate that EAT in patients with CAD is characterized by a unique expression pattern of adipokines and notably overexpressed adipokines related to inflammation.

### Adipokines and echocardiographic heart parameters

In the search for a relation between adipokines and heart disease, we created a regression model that included three independent variables: an adipokine plasma level or adipokine tissue expression, patient’s BMI, and patient’s age. Then we performed an analysis with a single echocardiographic heart parameter revealing a number of significant and independent relations. Protein content and mRNA expression of cytokines in SAT does not always reflect their plasma concentrations and hence it is difficult to conclude any effects on the heart. In order to create a clearer view on the observed relations, we focused on plasma and EAT adipokines only.

Impaired LV filling was associated with increased expressions of leptin in EAT, and elevated concentrations of resistin, leptin, and IL-6 in plasma. This is consistent with other studies [28–31]. Surprisingly, adiponectin plasma concentration was also related to LV diastolic dysfunction. It has been established that adiponectin is a cardioprotective protein [32]. Adiponectin shares signaling pathways with natriuretic peptides and therefore it could be related to heart remodeling. Lindberg et al. observed that increased plasma adiponectin concentrations were strongly related to the risk of HF [33]. There was also a positive correlation between apelin plasma concentration and E/A ratio, indicating that higher apelin levels were associated with better LV diastolic function. Consistently with our observations, Cheng et al. found decreased serum apelin levels in patients with diastolic heart failure [34].

In accordance with other studies, echocardiographic traits of heart hypertrophy were positively associated with resistin in plasma, and there was a negative correlation with plasma adiponectin [35, 36]. Associations with leptin were ambiguous, as one of the indices correlated positively, whereas the other one correlated negatively with leptin in EAT.

In the analysis we found that impairment in LV systolic function was related to an increase in EAT TNF-α mRNA indicating its potential role in HF. Other studies have already linked serum TNF-α with HF [37].

Interestingly, there was a clear difference in mRNA profile of adipokines in EAT between patients with HF and preserved or reduced EF. Normal EF was associated with lower TNF-α mRNA, elevated IL-6, and elevated visfatin mRNA. As we discovered in regression analysis, TNF-α in EAT is negatively correlated with EF, but we found no such correlations for IL-6 and visfatin. Both adipokines were previously linked to heart failure, but studies did not differentiate between systolic and diastolic HF. Increased blood levels of IL-6 were associated to systolic heart dysfunction [38]. Reduced function of visfatin in a study on a knock-out mouse model, was associated with a rapid progression of heart failure [39].

It is important to note that we found independent relations between echocardiographic heart parameters and some of the adipokine expressions in EAT. We found leptin and visfatin in EAT to be linked with cardiac remodeling. Left ventricle diastolic dysfunction was associated with IL-6 and leptin expression in EAT. Decrease in LV contractility correlated with TNF-α EAT expression. In the comparison of HFpEF and HFrEF patients we found significant differences in EAT expression of as much as three adipokines. Our results support the hypothesis, that despite its relatively small mass, EAT could have an impact on heart function through production of adipokines acting directly on cardiomyocytes. Obesity and diabetes have very limited effect on expression of adipokines in EAT in CAD patients. Possibly, local inflammation plays more important role in EAT adipokine balance. To the best of our knowledge this is the first study to evaluate expressions of adipokines in EAT with relation to echocardiographic parameters.

## Conclusions

The results of our study suggest that obesity and T2DM in individuals with CAD have a limited effect on plasma concentrations and fat tissue expressions of adipokines. Protein and mRNA expressions of adipokines in EAT seem to be more independent from obesity and T2DM, than in SAT. Moreover, the profile of adipokines in EAT differs from SAT, what might have an important influence on the heart. Here we report that EAT expressions of certain adipokines are associated to echocardiographic heart parameters, independently of BMI and age. Influence of circulating adipokines, especially leptin, resistin, and IL-6 must also be taken into consideration, as those were strongly related to diastolic function of the LV. The exact relation between adipokines and heart diseases remains elusive and requires further research.

## Supplementary information

**Additional file 1.** The panel shows representative blots for each protein: SAT is labeled with 1, EAT with 2. Four patients in each study group were selected randomly (12 in total). GAPDH was used as a loading control. EAT, epicardial adipose tissue; SAT, subcutaneous adipose tissue; IL-6, interleukin 6; GAPDH, Glyceraldehyde 3-phosphate dehydrogenase.

## Data Availability

The datasets used and/or analysed during the current study are available from the corresponding author on reasonable request.

## Abbreviations

EAT: epicardial adipose tissue
SAT: subcutaneous adipose tissue
VAT: visceral adipose tissue
IL-6: interleukin 6
TNF-α: tumor necrosis factor α
APN: adiponectin
CAD: coronary artery disease
T2DM: type 2 diabetes mellitus
BMI: body mass index
HOMA2-IR: The Homeostasis Model Assessment-Insulin Resistance
HbA1_c_: glycated haemoglobin
SBP: systolic blood pressure
DBP: diastolic blood pressure
HF: heart failure
HFrEF: heart failure with a reduced ejection fraction
HFpEF: heart failure with a preserved ejection fraction
LVH: left ventricular hypertrophy
NYHA: New York Heart Association
CCS: Canadian Cardiovascular Society
UCP-1: uncoupling protein 1
GAPDH: glyceraldehyde 3-phosphate dehydrogenase
CYCA: cyclophilin A
ELISA: enzyme-linked immunosorbent assay
SD: standard deviation
β: standardized regression coefficient
ANOVA: analysis of variance
LV: left ventricle
RV: right ventricle
LA: left atrium diameter
EATt: epicardial adipose tissue thickness
RVdD: right ventricle diameter in diastole
LVdD: left ventricular end-diastolic diameter
LVsD: left ventricular end-systolic diameter
LVM: left ventricular mass
LVMi: left ventricular mass index
IVSD: interventricular septum diastolic diameter
RWT: relative wall thickness
PWTd: posterior wall thickness in diastole
TAPSE: tricuspid annular plane systolic excursion
E/A: the ratio of peak velocity blood flow in early diastole to peak velocity flow in late diastole
E′: peak mitral annulus velocity in early diastole
A′: peak mitral annulus velocity in late diastole
DT′: deceleration time of the mitral annulus measured in tissue Doppler
FS: fractional shortening
EF: ejection fraction
DT: deceleration time
IVRT: isovolumic relaxation time

## References

[1] Halaas JL, Gajiwala KS, Maffei M, Cohen SL, Chait BT, Rabinowitz D (1995). Weight-reducing effects of the plasma protein encoded by the obese gene. Science.

[2] Mandviwala T, Khalid U, Deswal A (2016). Obesity and cardiovascular disease: a risk factor or a risk marker?. Curr Atheroscler Rep..

[3] Iacobellis G, Corradi D, Sharma AM (2005). Epicardial adipose tissue: anatomic, biomolecular and clinical relationships with the heart. Nat Clin Pract Cardiovasc Med..

[4] Sacks HS, Fain JN (2007). Human epicardial adipose tissue: a review. Am Heart J.

[5] Toczyłowski K, Gruca M, Baranowski M (2013). Epicardial adipose tissue and its role in cardiac physiology and disease. Postepy Hig Med Doswiadczalnej Online.

[6] Pandey G, Shihabudeen MS, David HP, Thirumurugan E, Thirumurugan K (2015). Association between hyperleptinemia and oxidative stress in obese diabetic subjects. J Diabetes Metab Disord..

[7] Park HK, Kwak MK, Kim HJ, Ahima RS (2017). Linking resistin, inflammation, and cardiometabolic diseases. Korean J Intern Med.

[8] Maggio M, Guralnik JM, Longo DL, Ferrucci L (2006). Interleukin-6 in aging and chronic disease: a magnificent pathway. J Gerontol A Biol Sci Med Sci.

[9] Ferrari R (1999). The role of TNF in cardiovascular disease. Pharmacol Res.

[10] Lee S, Kwak H-B (2014). Role of adiponectin in metabolic and cardiovascular disease. J Exerc Rehabil..

[11] Shibata R, Ouchi N, Ohashi K, Murohara T (2017). The role of adipokines in cardiovascular disease. J Cardiol..

[12] Spiroglou SG, Kostopoulos CG, Varakis JN, Papadaki HH (2010). Adipokines in periaortic and epicardial adipose tissue: differential expression and relation to atherosclerosis. J Atheroscler Thromb..

[13] Iacobellis G, Assael F, Ribaudo MC, Zappaterreno A, Alessi G, Di Mario U (2003). Epicardial fat from echocardiography: a new method for visceral adipose tissue prediction. Obes Res.

[14] Chechi K, Gelinas Y, Mathieu P, Deshaies Y, Richard D (2012). Validation of reference genes for the relative quantification of gene expression in human epicardial adipose tissue. PLoS ONE.

[15] de Simone G, Daniels SR, Devereux RB, Meyer RA, Roman MJ, de Divitiis O (1992). Left ventricular mass and body size in normotensive children and adults: assessment of allometric relations and impact of overweight. J Am Coll Cardiol.

[16] Tchernof A, Després J-P (2013). Pathophysiology of human visceral obesity: an update. Physiol Rev.

[17] Kouidhi S, Jarboui S, Clerget Froidevaux M-S, Abid H, Demeneix B, Zaouche A (2010). Relationship between subcutaneous adipose tissue expression of leptin and obesity in Tunisian patients. Tunis Med..

[18] Sindhu S, Thomas R, Shihab P, Sriraman D, Behbehani K, Ahmad R (2015). Obesity is a positive modulator of IL-6R and IL-6 expression in the subcutaneous adipose tissue: significance for metabolic inflammation. PLoS ONE.

[19] Cheng K-H, Chu C-S, Lee K-T, Lin T-H, Hsieh C-C, Chiu C-C (2005). Adipocytokines and proinflammatory mediators from abdominal and epicardial adipose tissue in patients with coronary artery disease. Int J Obes.

[20] Adela R, Reddy PNC, Ghosh TS, Aggarwal S, Yadav AK, Das B (2019). Serum protein signature of coronary artery disease in type 2 diabetes mellitus. J Transl Med..

[21] Mazurek T, Zhang L, Zalewski A, Mannion JD, Diehl JT, Arafat H (2003). Human epicardial adipose tissue is a source of inflammatory mediators. Circulation.

[22] Fain JN, Sacks HS, Bahouth SW, Tichansky DS, Madan AK, Cheema PS (2010). Human epicardial adipokine messenger RNAs: comparisons of their expression in substernal, subcutaneous, and omental fat. Metabolism..

[23] Hirata Y, Kurobe H, Akaike M, Chikugo F, Hori T, Bando Y (2011). Enhanced inflammation in epicardial fat in patients with coronary artery disease. Int Heart J..

[24] Zhou Y, Wei Y, Wang L, Wang X, Du X, Sun Z (2011). Decreased adiponectin and increased inflammation expression in epicardial adipose tissue in coronary artery disease. Cardiovasc Diabetol..

[25] Bambace C, Sepe A, Zoico E, Telesca M, Olioso D, Venturi S (2014). Inflammatory profile in subcutaneous and epicardial adipose tissue in men with and without diabetes. Heart Vessels.

[26] Vogel C, Marcotte EM (2012). Insights into the regulation of protein abundance from proteomic and transcriptomic analyses. Nat Rev Genet.

[27] Schwanhäusser B, Busse D, Li N, Dittmar G, Schuchhardt J, Wolf J (2011). Global quantification of mammalian gene expression control. Nature.

[28] Puurunen VP, Lepojärvi ES, Piira OP, Hedberg P, Junttila MJ, Ukkola O (2016). High plasma leptin levels are associated with impaired diastolic function in patients with coronary artery disease. Peptides.

[29] Dinh W, Füth R, Nickl W, Krahn T, Ellinghaus P, Scheffold T (2009). Elevated plasma levels of TNF-alpha and interleukin-6 in patients with diastolic dysfunction and glucose metabolism disorders. Cardiovasc Diabetol..

[30] Prins KW, Archer SL, Pritzker M, Rose L, Weir EK, Sharma A (2018). Interleukin-6 is independently associated with right ventricular function in pulmonary arterial hypertension. J Heart Lung Transplant Off Publ Int Soc Heart Transplant..

[31] Lebeche D (2015). Diabetic cardiomyopathy: is resistin a culprit?. Cardiovasc Diagn Ther..

[32] Aprahamian TR, Sam F (2011). Adiponectin in cardiovascular inflammation and obesity. Int J Inflamm..

[33] Lindberg S, Jensen JS, Bjerre M, Pedersen SH, Frystyk J, Flyvbjerg A (2014). Cardio-adipose tissue cross-talk: relationship between adiponectin, plasma pro brain natriuretic peptide and incident heart failure. Eur J Heart Fail.

[34] Chong KS, Gardner RS, Morton JJ, Ashley EA, McDonagh TA (2006). Plasma concentrations of the novel peptide apelin are decreased in patients with chronic heart failure. Eur J Heart Fail.

[35] Fontes-Carvalho R, Pimenta J, Bettencourt P, Leite-Moreira A, Azevedo A (2015). Association between plasma leptin and adiponectin levels and diastolic function in the general population. Expert Opin Ther Targets..

[36] Kim M, Oh JK, Sakata S, Liang I, Park W, Hajjar RJ (2008). Role of resistin in cardiac contractility and hypertrophy. J Mol Cell Cardiol.

[37] Feldman AM, Combes A, Wagner D, Kadakomi T, Kubota T, Li YY (2000). The role of tumor necrosis factor in the pathophysiology of heart failure. J Am Coll Cardiol.

[38] Raymond RJ, Dehmer GJ, Theoharides TC, Deliargyris EN (2001). Elevated interleukin-6 levels in patients with asymptomatic left ventricular systolic dysfunction. Am Heart J.

[39] Karamanlidis G, Lee CF, Garcia-Menendez L, Kolwicz SC, Suthammarak W, Gong G (2013). Mitochondrial complex I deficiency increases protein acetylation and accelerates heart failure. Cell Metab.

