# Supplementary figures and images for: Plasma concentration and expression of adipokines in epicardial and subcutaneous adipose tissue are associated with impaired left ventricular filling pattern
Source: J Transl Med. 2019 Sep 18;17:310. doi: 10.1186/s12967-019-2060-7 (PMC6751580; doi:10.1186/s12967-019-2060-7)

# Additional file 1

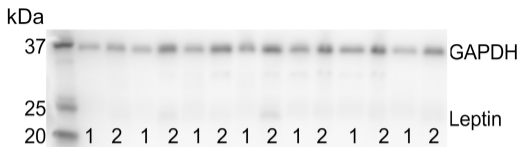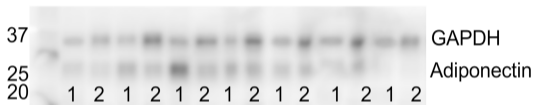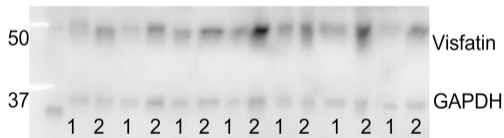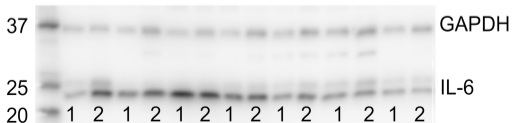

Supplement: Supplementary file 1 — Additional file 1. The panel shows representative blots for each protein: SAT is labeled with 1, EAT with 2. Four patients in each study group were selected randomly (12 in total). GAPDH was used as a loading control. EAT, epicardial adipose tissue; SAT, subcutaneous adipose tissue; IL-6, interleukin 6; GAPDH, Glyceraldehyde 3-phosphate dehydrogenase. [file 12967_2019_2060_MOESM1_ESM.pdf]
